# Hospital outcomes for patients with pulmonary arterial hypertension in sepsis and septic shock

**DOI:** 10.1186/s12890-022-02145-1

**Published:** 2022-10-03

**Authors:** Rhythm Vasudeva, Abhiram Challa, Nicholas Tuck, Suveeenkrishna Pothuru, Mohinder Vindhyal

**Affiliations:** 1 Internal Medicine/Paediatrics, Wesley Medical Center, University of Kansas School of Medicine-Wichita, Wichita, KS USA; 2 Department of Internal Medicine, Ascension Via Christi Hospital, Manhattan, KS USA; 3 Department of Cardiovascular Medicine, University of Kansas Medical Center, Kansas City, KS USA; 4 Internal Medicine, University of Kansas School of Medicine - Wichita, Wichita, KS USA

**Keywords:** Pulmonary hypertension, Sepsis, Septic shock, Hospital, Outcomes, Mortality

## Abstract

**Background:**

Pulmonary arterial hypertension (PAH) is associated with increased morbidity and mortality risk. The risk for adverse outcomes in patients with PAH in sepsis or septic shock (SSS) is uncertain.

**Methods:**

Adult patients diagnosed with SSS were identified in the National Readmissions Database over the years 2016–2017. A 2:1 ratio nearest propensity matching method was employed for several demographic, social, and clinical variables. In-hospital outcomes were compared between patients with PAH and those without, using t-test and chi-squared test as appropriate. Patients with cardiogenic shock were excluded. Relevant ICD-10 codes were used, and statistical significance was set at 0.05.

**Results:**

A total of 1,134 patients with PAH and sepsis/septic shock were identified, with a mean age of 65 years and 67% identifying as females. Patients with PAH had a higher prevalence of some chronic conditions, including chronic pulmonary disease, renal failure, congestive heart failure, coronary artery disease, obesity, coagulation disease. The prevalence of type 2 diabetes mellitus and alcohol use was lower in this cohort. After matching, patients with PAH and SSS, when compared to those with SSS and without PAH, had an increased occurrence of acute heart failure (24.1% vs. 19.6%, p = 0.003), amongst clinical outcomes. The differences in the occurrence of death, vasopressor use, paroxysmal atrial fibrillation, acute myocardial infarction, acute kidney injury, and stroke outcomes were not statistically different between the two groups. Patients with PAH, however, had a longer hospital stay (13.5 days vs. 10.9 days, p < 0.001) and hospital costs ($164,252 vs. $129,185, p < 0.001).

**Conclusion:**

Patients with PAH have worse outcomes for acute heart failure in sepsis or septic shock. Other mortality and morbidity outcomes are not statistically different. PAH is also associated with a longer hospital stay and increased hospital costs. These findings should be interpreted recognizing the inclusion of patients with re-admissions and the administrative nature of the database.

**Supplementary Information:**

The online version contains supplementary material available at 10.1186/s12890-022-02145-1.

## Introduction

Patients with pulmonary hypertension, especially with underlying comorbidities, have been shown to have reduced survival [1], with sepsis and septic shock being an especially precarious and challenging state with increased risk for decompensation [2]. Pulmonary artery hypertension (PAH) is a rare disease classified as a group 1 subset of pulmonary hypertension [3]. The survival time for patients with PAH has been historically low [4], but is improving with better therapy options available[5, 6].

Most outcome studies in PAH focus on right heart failure, [7] which have generally demonstrated worse outcomes. Infection is a significant cause of decompensated right heart failure with a subsequent increase in mortality [8]. However, another study contradicts these findings, showing no difference in 30-day mortality in sepsis or pneumonia for patients with PAH [9]. An independent correlation of sepsis and right ventricular strain independent of pulmonary hypertension has also been demonstrated in animal models [10], potentially suggesting a higher risk profile for patients with PAH in acute infectious processes.

The current literature lacks in establishing the impact of PAH in patients with sepsis or septic shock (SSS). This study aims to investigate in-hospital outcomes relating to mortality, morbidity, and resource utilization, for patients with PAH in the setting of sepsis or septic shock.

## Methods

### Data source

Data were retrieved from the Nationwide Readmissions Database (NRD) over 2016 and 2017. NRD is compiled from the Healthcare Cost and Utilization Project (HCUP) state in-patient databases, representing 58% of all US hospitalizations from 28 states. The HCUP conducts multiple quality assurance activities from various participating sites to verify the accuracy of the data. An Institutional Review Board approval was not necessary since the data is de-identified and publicly available.

### Participants

**Fig. 1:**
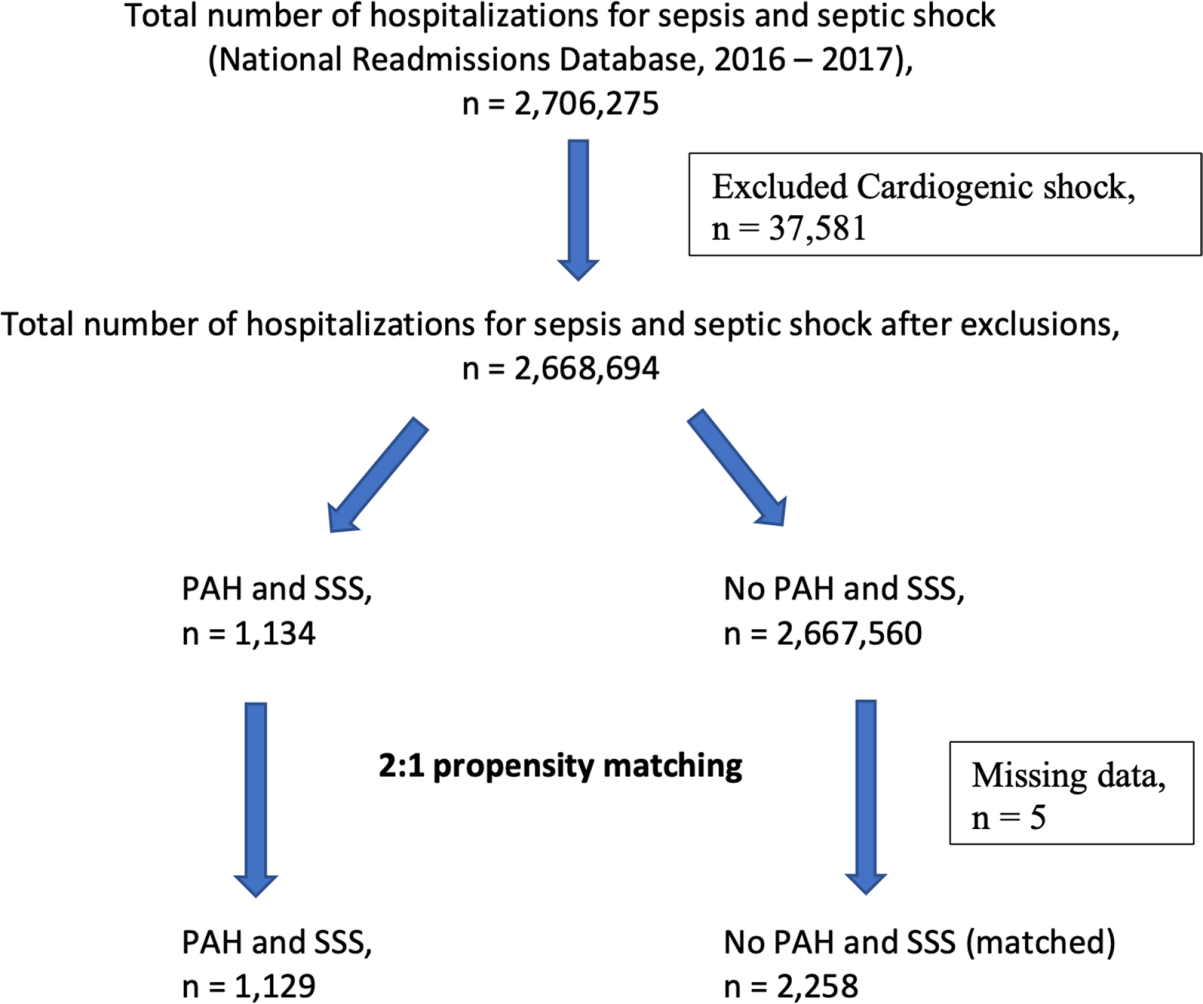
Derivation of study population for patients with pulmonary arterial hypertension and sepsis or septic shock. This figure demonstrates how the study population of patients with PAH and SSS was selected from the NRD. A total of 1,134 patients with PAH and SSS were identified over years 2016–2017 after applying the exclusion criteria. Five patients had data missing for the propensity matched variables, and were, subsequently, excluded from the final cohort for comparison. PAH = Pulmonary arterial hypertension. SSS = Sepsis or Septic Shock

All patients in this study were 18 years or older and received a diagnosis of sepsis or septic shock during their hospital stay using the International Classification of Diseases, 10^th^ edition [ICD-10] diagnosis codes. All patients with the diagnosis of PAH were identified. The relevant ICD-codes for these conditions are shown in Additional file 1: Table S1. Patients diagnosed with cardiogenic shock were excluded from the study. Additional exclusions included patients with pregnancy-related sepsis and septic shock diagnosis and patients who could not match the respective matching variables due to missing data. Figure 1 shows the study flowchart demonstrating derivation of the study population.

### Outcomes

The primary outcome of the study was to examine in-hospital mortality. Additional outcomes included vasopressor use, stroke outcomes, acute heart failure (systolic, diastolic, or combined), acute kidney injury, and resource utilization outcomes, including length of stay (LOS) and total hospital charges.

### Statistical analysis

All analyses were conducted with the weighted data using R software 4.0.5. Baseline characteristics were compared using t-test and chi-squared methods as appropriate prior to matching. We used clinical acumen to determine relevant demographic, social and clinical variables for propensity matching, with a 2:1 matching ratio. These included age, sex, hypertension, chronic pulmonary disease, renal failure, type 2 diabetes, coronary artery disease (CAD), chronic heart failure, obesity, coagulation disorders, anaemia, blood loss, electrolyte imbalance, drug abuse, alcohol abuse, elective admission status, and insurance status. In-hospital outcomes were compared for patients with sepsis or septic shock and PAH to those without PAH, using t-test and chi-squared methods as appropriate. A p-value of 0.05 was set for statistical significance.

## Results

A total of 2,668,694 patients were identified with a diagnosis of sepsis or septic shock during their hospital stay. Out of these, 1,134 patients were also found to have PAH with a mean age of 65 years and 67% identifying as females.

Patients with SSS and PAH compared to SSS without PAH had a significantly higher prevalence of some chronic conditions, including chronic pulmonary disease (40.2 vs. 28.4% p < 0.001), renal failure (34.6 vs. 25.2%, p < 0.001), congestive heart failure (CHF) (56.4 vs. 23.5%, p < 0.001), coronary artery disease (27.5 vs. 23.1%, p = 0.001), obesity (21.5% vs 17.5%, p < 0.001), and coagulation disease (24.2 vs. 14.8%, p < 0.001). However, the prevalence of type 2 diabetes mellitus (30.7 vs. 34.3%, p = 0.012) and alcohol use (2.9 vs. 5.0%, p = 0.001) was significantly lower in patients with PAH and SSS. Table 1 shows comparison of baseline characteristics between the two groups.

**Table 1:** showing the comparison of baseline characteristics for patients admitted with sepsis or septic shock by status of underlying pulmonary arterial hypertension (PAH)

	Overall	No PAH	PAH	p-value
n	2,668,694	2,667,560	1134	
Age, years (mean (SD))	65.33 (17.59)	65.33 (17.59)	65.43 (17.80)	0.841
Female, n (%)	1,320,965 (49.5)	1,320,209 (49.5)	756 (66.7)	< 0.001
Insurance pay, n (%)				< 0.001
Medicare	1,693,794 (63.5)	1,693,006 (63.5)	788 (69.5)	
Medicaid	380,324 (14.3)	380,207 (14.3)	117 (10.3)	
Private insurance	446,994 (16.7)	446,807 (16.7)	187 (16.5)	
Self-pay	72,725 (2.7)	72,708 (2.7)	17 (1.5)	
No charge	9297 (0.3)	9296 (0.3)	1 (0.1)	
Other	62,063 (2.3)	62,042 (2.3)	21 (1.9)	
NA	3497 (0.1)	3494 (0.1)	3 (0.3)	
Quartile for income by zip-code*, n (%)				0.015
1st (0–25th percentile)	772,173 (28.9)	771,884 (28.9)	289 (25.5)	
2nd (26th–50th percentile)	685,207 (25.7)	684,921 (25.7)	286 (25.2)	
3rd (51st–75th percentile)	646,050 (24.2)	645,755 (24.2)	295 (26.0)	
4th (76th–100th percentile)	525,348 (19.7)	525,095 (19.7)	253 (22.3)	
NA	39,916 (1.5)	39,905 (1.5)	11 (1.0)	
Elective admission, n (%)				0.837
No	2,576,629 (96.6)	2,575,533 (96.6)	1096 (96.6)	
Yes	88,808 (3.3)	88,772 (3.3)	36 (3.2)	
NA	3257 (0.1)	3255 (0.1)	2 (0.2)	
Hypertension, n (%)	1,710,022 (64.1)	1,709,389 (64.1)	633 (55.8)	< 0.001
Type 2 Diabetes, n (%)	914,654 (34.3)	914,306 (34.3)	348 (30.7)	0.012
Chronic pulmonary disorder, n (%)	758,656 (28.4)	758,200 (28.4)	456 (40.2)	< 0.001
Renal failure, n (%)	672,134 (25.2)	671,742 (25.2)	392 (34.6)	< 0.001
Congestive heart failure, n (%)	626,360 (23.5)	625,720 (23.5)	640 (56.4)	< 0.001
Coronary artery disease, n (%)	617,036 (23.1)	616,724 (23.1)	312 (27.5)	0.001
Obesity, n (%)	465,750 (17.5)	465,506 (17.5)	244 (21.5)	< 0.001
Coagulation disease, n (%)	396,378 (14.9)	396,104 (14.8)	274 (24.2)	< 0.001
Deficiency anaemia, n (%)	842,400 (31.6)	842,025 (31.6)	375 (33.1)	0.29
Blood loss anaemia, n (%)	30,373 (1.1)	30,361 (1.1)	12 (1.1)	0.909
Depression, n (%)	368,515 (13.8)	368,347 (13.8)	168 (14.8)	0.348
Fluid electrolyte imbalance, n (%)	1,590,019 (59.6)	1,589,311 (59.6)	708 (62.4)	0.054
Drug abuse, n (%)	167,747 (6.3)	167,676 (6.3)	71 (6.3)	1
Alcohol abuse, n (%)	133,805 (5.0)	133,772 (5.0)	33 (2.9)	0.001

*Income range for quartiles in 2016 and 2017:

1st Quartile: $1–$42,999 in 2016 and $1–$43,999 in 2017,

2nd Quartile: $43,000–$53,999 in 2016 and $44,000–$55,999 in 2017,

3rd Quartile: $54,000–$70,999 in 2016 and $56,000–$73,999 in 2017,

4th Quartile: $71,000 + in 2016 and $74,000 + in 2017

**Fig. 2:**
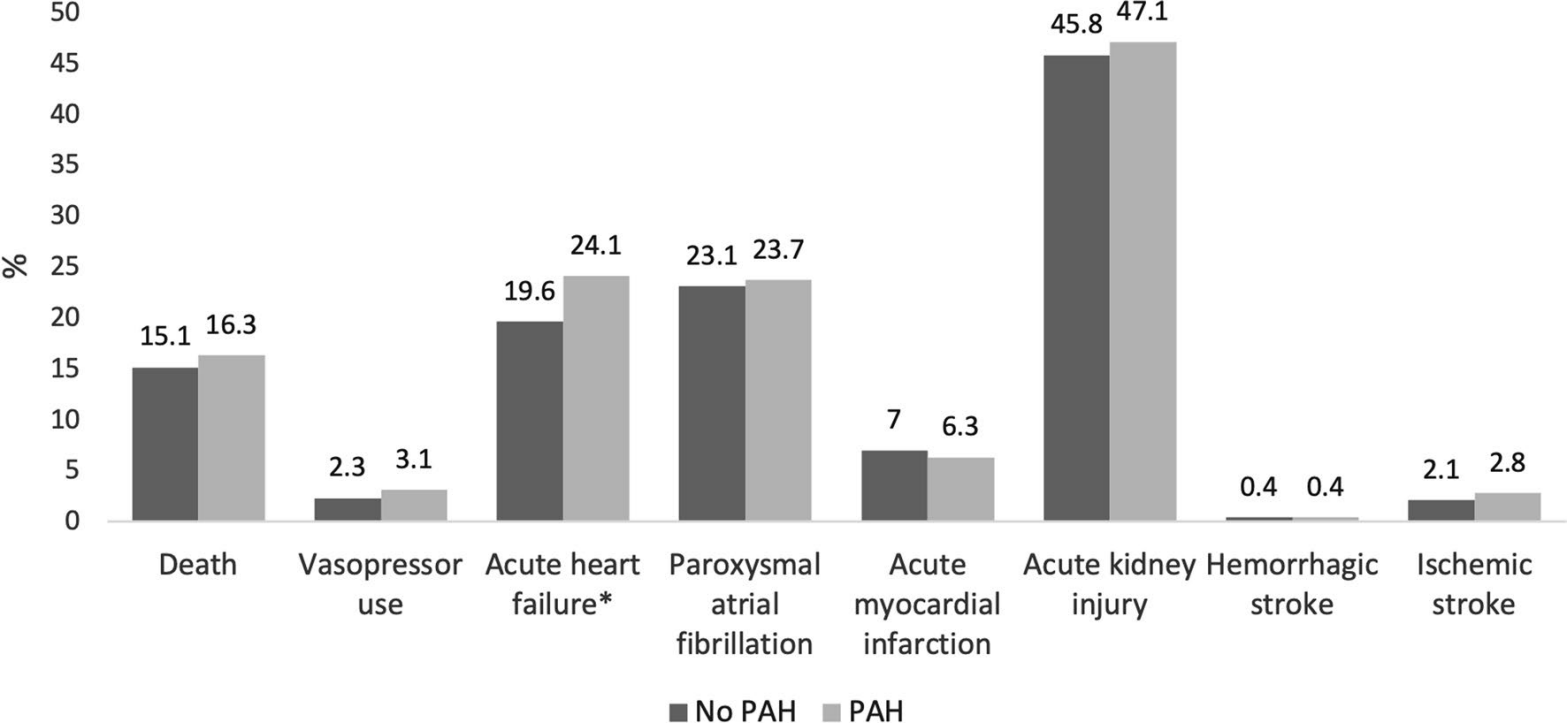
Differences in in-hospital clinical outcomes for patients with and without PAH in SSS. This figure highlights the impact of pulmonary arterial hypertension on in-hospital outcomes for patients admitted with sepsis or septic shock. Pulmonary arterial hypertension was significantly associated with an increased occurrence for acute heart failure. *Significant p-value (p = 0.003) PAH = Pulmonary arterial hypertension. SSS = Sepsis or Septic Shock

Upon matching with complete data on matching variables, 1,129 patients were identified with SSS and PAH. When comparing their in-hospital outcomes with patients with SSS and without PAH, the difference in the primary outcome, death, was not significant (16.3 vs. 15.1%, p = 0.41), respectively. In terms of secondary outcomes, the SSS and PAH cohort had significantly higher rates of acute heart failure (24.1 vs. 19.6%, p < 0.003), length of stay (13.5 days vs 10.9 days, p < 0.001), and hospital costs (($164,252 vs. $129,185, p < 0.001). Other secondary outcomes that showed no significant differences between the two cohorts were vasopressor use (3.1 vs. 2.3%, p < 0.236), atrial fibrillation (23.7 vs. 23.1%, p < 0.698), acute myocardial infarction (6.3 vs. 7.0%, p < 0.454), acute kidney injury (47.1 vs. 45.8%, p < 0.503), haemorrhagic strokes (0.4 vs. 0.4%, p = 1) and ischemic strokes (2.8 vs. 2.1%, p < 0.246). These differences are presented in Fig. 2 and Table 2.

**Table 2:** showing the differences in resource utilization outcomes for patients with and without pulmonary arterial hypertension (PAH) in sepsis or septic shock

	No PAH	PAH	p-value
Length of stay in days, mean (SD)	10.9 (13.4)	13.5 (20.4)	< 0.001
Hospital costs in $, mean (SD)	129,184.55 (216,367.74)	164,252.24 (343,013.71)	< 0.001

## Discussion

Our results indicate that patients with PAH do not have a higher risk of worse clinical outcomes when admitted for sepsis or septic shock, except for acute heart failure. However, patients with PAH had worse resource utilization outcomes, including length of stay and hospitalization costs.

Patients with PAH are a vulnerable group, especially above the age of 50 years, since they have greater comorbidities [11], particularly relating to the heart. This is confirmed in our cohort of patients, whereby patients with PAH had a history of CHF by more than 30% compared to other patients with SSS without PAH. Given such predilection to structural heart disease in patients with PAH, their risk for decompensation is significantly greater given the limitation in meeting the body's perfusion demands in septic shock [2]. Our findings reflect such risk of decompensation with a higher occurrence of acute heart failure in patients with PAH who get admitted for SSS. These findings are supported by existing literature, which demonstrates that infection is associated with a worse prognosis for acute heart failure in patients with PAH [12]. Critical care for such patients requires adequate treatment of the cause for the decompensation, such as sepsis, and optimizing the right ventricular preload and afterload and right ventricular inotropy [13]. This is especially important since the long-term survival for patients with pulmonary hypertension after hospitalization for their first acute right heart failure has shown to be low (41.9% at 2-years) [7].

Despite a higher predilection for and worse prognosis of acute heart failure, our findings indicate that patients with PAH and SSS did not have significantly worse in-hospital clinical outcomes, including mortality. This supplements some existing literature which showed that PAH was not associated with increased 30-day mortality in patients admitted for sepsis (odds ratio 0.93, 95% confidence interval (CI) 0.59–1.44) [9]. In a non-comparative study, sepsis was identified as the most common cause of non-cardiac-related hospitalizations (25%) for patients with PAH [14]. It was also reported that mortality in patients with PAH was worse when admitted for non-cardiac causes versus a primary cardiac hospitalization (6.9 vs. 5.3%). Therefore, hospitalization and mortality burden for sepsis in patients with PAH is an important consideration, and several factors, including risk for decompensation and right ventricular strain, should be considered for optimal care of such patients [15]. In addition, our results add to the paucity of literature investigating additional hospital outcomes for patients with PAH. Other cardiovascular outcomes, including strokes, paroxysmal atrial fibrillation, and acute myocardial infarction, were comparable in SSS patients with and without PAH.

PAH has been demonstrated to have a greater economic burden relating to increased hospital visits and greater costs [16–18], compared to other chronic diseases [19]. Our findings confirm that this added burden for patients with PAH extends to those admitted with SSS since they had more extended hospital stays and increased hospitalization costs than those without PAH. A retrospective study in Spain demonstrated that expenses relating to PAH hospital admission have been on the rise between the years 2004 and 2015 [20]. These findings indicate the importance of investigating effective strategies in the management of PAH, especially in acute illness such as sepsis, to mitigate its burden on the healthcare system. Close follow-up for patients with PAH is an important strategy and has been shown to reduce medical costs [19].

The findings of this study should be interpreted with caution, given the following limitations. The administrative nature of the database makes the occurrence of ICD-10 billing coding errors possible, and thus, non-differential and misclassification biases cannot be excluded. However, HCUP has several mechanisms to ensure the validity of the data available [21]. Despite thorough efforts to appropriately match comparison groups based on comorbidities and demographic factors, several factors could not be accounted for, including the hemodynamic parameters, laboratory findings, and in-patient medications. In addition, the database also included patients with re-admissions within the same calendar year, which were regarded as distinct patient points in the analysis. Given the independent nature of the sepsis-related conditions, this is not expected to alter the interpretation of the findings relating to outcomes but may overestimate the actual patient burden. Lastly, data on ethnic and racial disparities were not provided in the dataset and, thus, any related differences remain unexplored.

When admitted for sepsis or septic shock, patients with pulmonary arterial hypertension had an increased occurrence of acute heart failure. Other clinical outcomes, including mortality, were not significantly different. However, these admissions were associated with worse resource utilization outcomes, including length of hospital stay and hospitalization costs. The careful in-patient management for patients with PAH, with special consideration to underlying right ventricular strain, is essential to optimize outcomes in acute states, such as sepsis and septic shock.

## Supplementary Information

**Additional file 1.** Supplementary Table 1.

## Data Availability

The dataset analysed during the current study are available in the “Healthcare Cost and Utilization Project—Nationwide Readmissions Database (HCUP-NRD)” repository, https://www.hcup-us.ahrq.gov/tech_assist/centdist.jsp. [Data available from HCUP].

## Abbreviations

PAH: Pulmonary arterial hypertension
SSS: Sepsis or septic shock
LOS: Length of stay
NRD: Nationwide Readmissions Database
HCUP: Healthcare Cost and Utilization Project
ICD: International Classification of Diseases
