# Supplementary material for: Hospital outcomes for patients with pulmonary arterial hypertension in sepsis and septic shock
Source: BMC Pulm Med. 2022 Oct 3;22:374. doi: 10.1186/s12890-022-02145-1 (PMC9528158; doi:10.1186/s12890-022-02145-1)
Supplement: Supplementary file 1 — Additional file 1. Supplementary Table 1. [file 12890_2022_2145_MOESM1_ESM.pdf]

Supplementary table 1 showing the relevant ICD-10 codes employed to identify patients with sepsis, septic shock and pulmonary arterial hypertension

Diagnosis		ICD-10 code
**Pulmonary arterial hypertension**	World Health Organization Classification Group 1	I27.0
**Sepsis/ Septic shock**	Salmonella sepsis	A02.1
	Anthrax sepsis	A22.7
	Erysipelothrix sepsis	A26.7
	Listerial sepsis	A32.7
	Sepsis due to streptococcus, group A	A40.0
	Sepsis due to streptococcus, group B	A40.1
	Sepsis due to Streptococcus pneumoniae	A40.3
	Other streptococcal sepsis	A40.8
	Streptococcal sepsis, unspecified	A40.9
	Sepsis due to Methicillin susceptible Staphylococcus aureus	A41.01
	Sepsis due to Methicillin resistant Staphylococcus aureus	A41.02
	Sepsis due to other specified staphylococcus	A41.1
	Sepsis due to unspecified staphylococcus	A41.2
	Sepsis due to Hemophilus influenzae	A41.3
	Sepsis due to anaerobes	A41.4
	Gram-negative sepsis, unspecified	A41.50
	Sepsis due to Escherichia coli [E. coli]	A41.51
	Sepsis due to Pseudomonas	A41.52
	Sepsis due to Serratia	A41.53
	Other Gram-negative sepsis	A41.59
	Sepsis due to Enterococcus	A41.81
	Other specified sepsis	A41.89
	Sepsis, unspecified organism	A41.9
	Actinomycotic sepsis	A42.7
	Gonococcal sepsis	A54.86
	Severe sepsis without septic shock	R65.20
	Severe sepsis with septic shock	R65.21
	Sepsis following a procedure, initial encounter	T81.44XA
	Sepsis following a procedure, subsequent encounter	T81.44XD
	Sepsis following a procedure, sequela	T81.44XS
	Postprocedural septic shock, initial encounter	T81.12XA
	Postprocedural septic shock, subsequent encounter	T81.12XD
	Postprocedural septic shock, sequela	T81.12XS
